# Editorial: Fusarium species as plant and human pathogens, mycotoxin producers, and biotechnological importance

**DOI:** 10.3389/ffunb.2023.1320198

**Published:** 2023-11-14

**Authors:** Sofia N. Chulze, Sheryl Tittlemier, Adriana M. Torres

**Affiliations:** ^1^ Research Institute on Mycology and Mycotoxicology (IMICO) National University of Rio Cuarto(UNRC) National Technological and Research Council from Argentina (CONICET), Rio Cuarto, Córdoba, Argentina; ^2^ Canadian Grain Commission, Grain Research Laboratory, Winnipeg, MB, Canada

**Keywords:** antifungal compounds, Fusarium, biocontrol, fusarium head blight, mycotoxins, maize, tobacco

The fungal genus *Fusarium* Link includes many plant pathogens of agricultural crops, human pathogens, and species with biotechnological applications (Leslie and Summerell, 2006; Aoki et al., 2014; Meyer et al., 2020; Geiser et al., 2021). The main concern in the last decades has been devoted to those species that infect staple crops and produce secondary metabolites known as mycotoxins. Mycotoxins produced by *Fusarium* species have been shown to occur worldwide. Among these, fumonisins and trichothecenes are of great concern for their impact on human and animal health (Munkvold et al., 2021). Under a scenario of climate change, the situation can worsen due to changes in fungal biodiversity, in the resistance/resilience of crops, and in the strong impact of the environmental factors affecting both global food security and safety (Singh et al., 2023).

This Research Topic describes recent advances in the field of plant pathogens and toxigenic *Fusarium* species and strategies to reduce their impact.

In this Research Topic, four works (three original research articles and one hypothesis and theory article) were published on *Fusarium* species that are important as plant pathogens in maize, wheat, and tobacco and the recent advances in their biodiversity and control.

Environmentally friendly strategies are explored to reduce the impact of mycotoxins produced by *Fusarium* species on cereals (Petrucci et al., 2023). The manuscript of Satterlee et al., on the transcriptomic response of *Fusarium verticillioides* to variably inhibitory environmental isolates of *Streptomyces*, described the evaluation of *Streptomyces* strains to control *F. verticillioides.* The strains that showed more impact on *F. verticillioides* growth also caused more impact in the transcriptomic response. Variation in time response was observed. Among the genes involved, one was related to nitrogen assimilation and others to the gene cluster of fusaric acid. This metabolite is important in the interaction of *F. verticillioides* with potential antagonists. Other genes affected were those related to β lactamases produced by *F. verticillioides*; these enzymes were induced under the confrontation of the pathogen with *Streptomyces* strains. The potential of *Streptomyces* species as biocontrol agents against *F. verticillioides* was highlighted.

Wheat (*Triticum aestivum* L and *T. durum* L) is a global staple food that is susceptible to infection by various *Fusarium* species. Under certain conditions, *Fusarium* infection can lead to *Fusarium head blight* (FHB), one of the most critical fungal diseases of wheat. In epidemic years, FHB decreases grain yield and quality and impacts the safety of wheat due to the production of mycotoxins. The trichothecene mycotoxin deoxynivalenol (DON) is of particular concern due to its potential health impacts and regulated status in many jurisdictions. The occurrence and severity of FHB are governed by several factors, including the aggressiveness and mycotoxin production potential of the infecting *Fusarium* species.

The work done by Bamforth et al. surveyed the presence of various *Fusarium* species in Canadian wheat harvest samples. They examined the relationships of *Fusarium* species, including the trichothecene chemotype, present on visually identified *Fusarium*-damaged kernels with growing location, harvest year, and wheat species (i.e., hexaploid and durum wheat). They noted some biodiversity of *Fusarium* species across Canada in wheat grain, the most common species and genotype isolated being *F. graminearum* and 3-ADON, respectively. The occurrence of the 3-ADON genotype increased, particularly in the western Prairie regions, over the time period of the survey (2014-2020). The researchers also noted higher severity of FHB and DON accumulation in durum wheat compared to hexaploid wheat.

Tobacco (*Nicotiana tabacum* L.) is one of the most widely cultivated crops, produced in more than 125 countries worldwide, and it can be infected by *Fusarium species* within the *Fusarium oxysporum* and *F. solani* complexes, causing root rot (Food and Agriculture Organization-FAO, 2022).

Li et al. described the interactions between *Fusarium* and other members of the soil microbial community. The authors used high-throughput sequencing technology and found that infected soil had higher levels of soil nutrients but lower observed richness within the different microbial groups compared to healthy soil. The infection by *F. solani* had a significant impact on the microbial community structure and interactions in soil. Moreover, *F. solani* had a higher number of connecting nodes in infected soils. The study highlighted the importance of understanding the interactions among microorganisms in the soil ecosystem and the vulnerability of soil microbial communities to pathogen invasion.

Maize (*Zea mays* L) is grown in different regions and can also be contaminated with *Fusarium* species, including the trichothecene producers associated with red ear rot (*Fusarium graminearum* and related species) and the fumonisin producers associated with pink ear rot (*Fusarium verticillioides* and other species within the *Fusarium fujikuroi* complex). The contamination with these trichothecene and fumonisin mycotoxins causes toxic effects both to humans and animals and economic losses in the maize food and feed chains (Logrieco et al., 2021).

Cereals including maize, wheat, and rice contain compounds such as 2-benzoxazolinone that have shown antifungal activity. Gao et al. reported the ability of *Fusarium verticillioides* to degrade compounds that contain lactam and/or lactone moieties. Using transcriptome analysis, it was observed that besides the two previously identified gene clusters, FDB1 and FDB2, this fungal species degrades 2-benzoxazolinone when it is exposed to 2-benzoxazolinone and three related chemical compounds, 2-oxindole, 2-coumaranone, and chlorzoxazone. This degradation is mediated when other gene clusters are activated, including a cluster responsible for pyridoxine (vitamin B6) biosynthesis and the gene cluster for fusaric acid, among others. This study provides more data on the ability of *Fusarium verticillioides* to respond to stress conditions due to the presence of phytochemicals with antifungal activity.

There is still a variety of research being performed on *Fusarium* species, highlighting the relevance of the species within the genus as significant pathogens for agricultural crops. A better understanding of the roles of *Fusarium* species within their environment and their varied internal functions will help to manage this genus’s impact on the yield, quality, and safety of crops. Furthermore, more studies on the biotechnological potential of *Fusarium* species are encouraged.

## Author contributions

SC: Conceptualization, Writing – original draft. ST: Writing – review & editing. AT: Writing – review & editing.
